# Associations of metabolic dysfunction-associated steatotic liver disease and cardiometabolic burden with urinary tubular biomarker abnormalities: a cross-sectional health examination study

**DOI:** 10.3389/fmed.2026.1987414

**Published:** 2026-10-29

**Authors:** Li Yuan, Huimin Yin, Weimin Chen, Yangtian Wang, Dan Xiang

**Affiliations:** 1 Nanjing University Medical School Affiliated Taikang Xianlin Drum Tower Hospital, Nanjing, China; 2 School of Chinese Medicine, Nanjing University of Chinese Medicine, Nanjing, China

**Keywords:** cardiometabolic burden, health examination, kidney, MASLD, metabolic dysfunction, urinary tubular biomarkers

## Abstract

**Background:**

Metabolic dysfunction-associated steatotic liver disease (MASLD) frequently coexists with cardiometabolic abnormalities and has been associated with kidney disease. However, its relationship with urinary markers of tubular dysfunction and the extent to which this association overlaps with accompanying cardiometabolic burden remain unclear. We examined the associations of MASLD and cumulative cardiometabolic burden with urinary tubular biomarker abnormalities in a health-examination population.

**Methods:**

This cross-sectional study included 7,011 adults undergoing routine health examinations between January 2020 and December 2023. MASLD was defined by ultrasonographically detected hepatic steatosis in the presence of at least one cardiometabolic risk criterion. Urinary N-acetyl-*β*-D-glucosaminidase (uNAG), *α*1-microglobulin (uA1M), *β*2-microglobulin (u*β*2M), and retinol-binding protein (uRBP) were evaluated. The primary outcome was a composite urinary tubular biomarker abnormality, defined as at least one abnormal urinary biomarker. A cardiometabolic burden score ranging from 0 to 5 was constructed from adiposity, elevated blood pressure, dysglycemia, hypertriglyceridemia, and low high-density lipoprotein cholesterol. Multivariable logistic regression with sequential covariate adjustment was used to examine the association of MASLD with the composite outcome. Cardiometabolic burden was analyzed separately without adjustment for its constituent components.

**Results:**

Among 7,011 participants, 3,552 (50.7%) were classified as having MASLD. MASLD was associated with greater odds of the composite urinary biomarker abnormality after adjustment for age and sex (odds ratio [OR], 1.24; 95% confidence interval [CI], 1.11–1.39; *P* < 0.001). This association was attenuated after additional adjustment for measured cardiometabolic and renal characteristics (OR, 1.02; 95% CI, 0.89–1.17; *P* = 0.785). In separate analyses, each one-point increase in cardiometabolic burden was associated with higher odds of the composite urinary biomarker abnormality after adjustment for age and sex (OR, 1.21; 95% CI, 1.16–1.26; *P* < 0.001), with similar results after additional adjustment for estimated glomerular filtration rate (OR, 1.22; 95% CI, 1.16–1.27; *P* < 0.001). Greater cardiometabolic burden was also associated with higher concentrations of uNAG, uA1M, u*β*2M, and uRBP in age- and sex-adjusted analyses.

**Conclusions:**

In this cross-sectional health-examination cohort, the association between MASLD and urinary tubular biomarker abnormalities was substantially attenuated after conditioning on measured cardiometabolic characteristics, whereas greater cumulative cardiometabolic burden remained consistently associated with urinary biomarker abnormalities. These findings demonstrate substantial statistical overlap between MASLD and cardiometabolic risk but do not establish mediation or causality. Prospective studies using standardized urine collection and concentration-normalized biomarker measurements are needed to determine the temporal and prognostic significance of these associations.

## 1 Introduction

Metabolic dysfunction-associated steatotic liver disease (MASLD) was introduced in 2023 through a multisociety Delphi consensus to replace the previous terminology of non-alcoholic fatty liver disease (NAFLD) and to more explicitly incorporate the close relationship between hepatic steatosis and cardiometabolic dysfunction (1–3). Under the new framework, MASLD is defined by the presence of hepatic steatosis together with at least one cardiometabolic risk factor, including abnormalities in adiposity, glucose regulation, blood pressure, triglycerides, or high-density lipoprotein cholesterol (1–3). The change in nomenclature is more than semantic: it reframes steatotic liver disease within a broader cardiometabolic context and highlights the substantial overlap between hepatic steatosis, obesity, insulin resistance, dyslipidemia, and hypertension (1–3). Epidemiological studies using the earlier NAFLD definition estimated that steatotic liver disease affects approximately one-third of the global adult population (4, 5), while studies applying the new consensus nomenclature similarly demonstrate a high population burden of MASLD. A recent meta-analysis of 44 studies comprising more than 11 million individuals estimated a pooled global MASLD prevalence of approximately 33.6%, although prevalence varies substantially by geographic region, population characteristics, metabolic risk, and diagnostic modality (6).

The clinical relevance of MASLD extends beyond progressive liver disease. NAFLD and MASLD are increasingly considered systemic cardiometabolic phenotypes associated with cardiovascular disease, type 2 diabetes, and kidney disease (7–10). A substantial body of epidemiological evidence has linked NAFLD with prevalent and incident chronic kidney disease (CKD), albuminuria, and reduced estimated glomerular filtration rate (eGFR) (11–15). Earlier meta-analyses demonstrated that individuals with NAFLD had a higher incidence of CKD even after adjustment for several conventional renal risk factors (11–13), and more recent evidence using metabolic dysfunction-based liver disease definitions has generally supported an association with CKD (16). A contemporary meta-analysis of MASLD and CKD further illustrates the increasing interest in the coexistence of metabolic liver and kidney disease under the new nomenclature (17). These observations have stimulated interest in the potential liver–kidne*y* axis, for which insulin resistance, systemic low-grade inflammation, endothelial dysfunction, oxidative stress, altered lipid metabolism, neurohormonal activation, and ectopic lipid deposition have all been proposed as shared biological pathways (8–10, 18–21).

Nevertheless, most previous studies examining renal involvement in steatotic liver disease have focused on conventional kidney outcomes, including eGFR decline, albuminuria, or clinically defined CKD (11–17). These measures are clinically important and should not be regarded simply as “late” manifestations of kidney disease; however, they predominantly characterize glomerular filtration or albumin handling and may not fully capture abnormalities in tubular physiology (22–24). This distinction may be particularly relevant in metabolic disorders. The proximal tubule is highly metabolically active and has substantial energy requirements because of its central role in the reabsorption of glucose, electrolytes, amino acids, and low-molecular-weight proteins (20, 21, 25). Experimental and clinical evidence indicates that hyperglycemia, insulin resistance, lipid overload, mitochondrial dysfunction, and oxidative stress can alter proximal tubular cellular metabolism and function (18–21, 25). In diabetes and other metabolic disorders, urinary tubular markers have consequently been investigated as complementary indicators of renal tubular stress or dysfunction (22–27). However, the relationship between MASLD and these urinary tubular phenotypes has been much less extensively investigated than its relationship with conventional CKD outcomes.

Several urinary biomarkers capture different aspects of proximal tubular physiology and therefore require careful interpretation. Urinary N-acetyl-*β*-D-glucosaminidase (uNAG) is a lysosomal enzyme highly enriched in proximal tubular epithelial cells; increased urinary activity has been used as an indicator of tubular epithelial stress or injury in metabolic, toxic, and diabetic kidney disorders (22, 26, 27). In contrast, urinary *α*1-microglobulin (uA1M), *β*2-microglobulin (u*β*2M), and retinol-binding protein (uRBP) are low-molecular-weight proteins that are freely filtered at the glomerulus and normally reabsorbed in the proximal tubule. Increased urinary excretion therefore primarily reflects impaired proximal tubular reabsorptive capacity, although the performance of individual markers may also be influenced by urinary pH, protein stability, glomerular filtration, specimen handling, assay characteristics, and urine concentration (23, 24, 28–30). Importantly, these markers are not interchangeable measures of histological tubular injury. A composite abnormality across multiple urinary markers should therefore be interpreted as a urinary tubular biomarker phenotype, rather than as definitive evidence of structural tubular injury. This distinction is especially important in cross-sectional observational studies without renal histology or longitudinal kidney outcomes.

A second and conceptually distinct issue concerns the role of cardiometabolic burden. Under the MASLD framework, cardiometabolic abnormalities are integral to the exposure definition itself (1–3). Obesity, elevated blood pressure, impaired glucose regulation, hypertriglyceridemia, and reduced HDL-C are simultaneously established correlates of kidney dysfunction and have been associated with CKD both individually and in aggregate (31–33). Studies of metabolic syndrome have shown that increasing numbers of metabolic abnormalities are associated with progressively greater prevalence and incidence of CKD (31–33). Accordingly, MASLD and cumulative cardiometabolic burden should not be regarded as statistically independent constructs. Adjustment for the same cardiometabolic factors used to define MASLD may substantially alter the MASLD coefficient, but such attenuation cannot distinguish among confounding, exposure-definition overlap, overadjustment, non-collapsibility of the odds ratio, or a true biological pathway. It therefore does not by itself establish mediation, nor does it demonstrate that hepatic steatosis has no independent biological relevance. A complementary approach is to examine cumulative cardiometabolic burden separately, while avoiding adjustment for the same variables used to construct that burden score.

Against this background, several questions remain unresolved. First, although the relationship of NAFLD/MASLD with CKD has been extensively studied, much less is known about its association with a phenotype defined by multiple urinary markers reflecting tubular stress and proximal tubular reabsorptive dysfunction. Second, because MASLD inherently incorporates cardiometabolic abnormalities, the extent to which its association with urinary tubular biomarker abnormalities changes after conditioning on those characteristics requires careful interpretation. Third, it remains clinically and biologically relevant to determine whether the cumulative burden of cardiometabolic abnormalities, considered separately from MASLD classification, is associated with urinary tubular biomarker abnormalities. We therefore conducted a cross-sectional study of 7,011 adults undergoing routine health examinations. The primary objective was to examine the association between MASLD and a composite urinary tubular biomarker abnormality defined by uNAG, uA1M, u*β*2M, and uRBP. We additionally evaluated how the MASLD association changed across sequential covariate-adjustment models and, in a separate analysis, examined the association of cumulative cardiometabolic burden with the composite phenotype and individual urinary biomarkers without adjusting the burden score for its constituent components. Finally, exploratory subgroup analyses were performed to assess potential heterogeneity across clinically relevant characteristics. Given the cross-sectional design, all analyses were intended to characterize statistical associations rather than temporal, causal, or mediating relationships.

## 2 Materials and methods

### 2.1 Study design and population

This cross-sectional study was conducted among adults undergoing routine health examinations at Taikang Xianlin Drum Tower Hospital, Nanjing, China, between January 2020 and December 2023. Participants were health-examination attendees at our institution rather than individuals recruited through a population-based probability sampling framework. The routine examination program included demographic and anthropometric assessment, blood pressure measurement, fasting biochemical testing, abdominal ultrasonography, and urinary laboratory testing.

Participants were eligible for the present analysis if they had available abdominal ultrasonography, the demographic and biochemical variables required for MASLD classification and covariate assessment, and sufficient urinary biomarker information to evaluate the prespecified urinary tubular biomarker outcomes. The final analytical cohort comprised 7,011 unique participants. Each participant contributed only one health-examination record, and no repeated examinations from the same individual were included; therefore, observations were treated as independent at the participant level.

The archived analytical dataset contained the final eligible cohort used for this study. Reliable record-level counts of all health-examination attendees screened before creation of the analytical dataset and the exact numbers excluded at each pre-extraction stage were not retained in the archived research file. We therefore did not retrospectively reconstruct unverified exclusion counts. Within the final cohort, the demographic, anthropometric, biochemical, ultrasonographic, and clinical variables required for the primary analyses were available. Urinary biomarker measurements reported below the laboratory quantitative reporting limit were distinguished from conventional missing values and were handled separately in biomarker-specific analyses, as described below.

Because the study population was derived from a single-center health-examination population rather than from population-based random sampling, the cohort should not be considered representative of the general population. This sampling characteristic was taken into account when interpreting the external validity of the findings.

The studies involving human participants were reviewed and approved by the Ethics Committee of Taikang Xianlin Drum Tower Hospital (Approval No. LS202319). The study was conducted in accordance with the Declaration of Helsinki and relevant institutional and national regulations. The requirement for written informed consent was waived by the ethics committee because this retrospective study used routinely collected health examination data, involved no intervention, and did not include identifiable personal information.

### 2.2 Assessment of metabolic dysfunction-associated steatotic liver disease

Metabolic dysfunction-associated steatotic liver disease (MASLD) was defined in accordance with the 2023 multisociety Delphi consensus nomenclature (1). MASLD required the presence of hepatic steatosis together with at least one cardiometabolic risk criterion.

Hepatic steatosis was assessed by abdominal ultrasonography performed as part of the routine health examination. Ultrasonographic steatosis was identified on the basis of characteristic sonographic features, including increased hepatic echogenicity relative to the renal cortex, attenuation of ultrasound penetration, and reduced visualization of intrahepatic vascular structures. Ultrasonographic examinations were performed by trained clinical ultrasonography personnel according to routine institutional procedures. Because the study was retrospective, device-specific and operator-specific metadata were not consistently retained in the analytical dataset and could not be reliably reconstructed.

For the present study, the cardiometabolic criteria used for operational MASLD classification were based on the available measured variables in the health-examination dataset. A participant with ultrasonographically detected hepatic steatosis was classified as having MASLD if at least one of the following criteria was present:

1. Adiposity: body mass index (BMI) ≥ 23.0 kg/m2;
2. Dysglycemia: fasting plasma glucose ≥5.6 mmol/L or glycated hemoglobin (HbA1c) ≥ 5.7%;
3. Elevated blood pressure: systolic blood pressure ≥130 mmHg or diastolic blood pressure ≥85 mmHg;
4. Hypertriglyceridemia: triglycerides ≥1.70 mmol/L;
5. Low high-density lipoprotein cholesterol (HDL-C): HDL-C ≤ 1.0 mmol/L in men or ≤1.3 mmol/L in women.

Participants who did not meet this operational definition were classified as non-MASLD for the primary binary comparison. Accordingly, the non-MASLD group included both participants without ultrasonographically detected steatosis and a small number of participants with steatosis who did not meet any of the available cardiometabolic criteria. We therefore did not interpret the non-MASLD group as synonymous with a metabolically healthy reference population.

Information on the use of antihypertensive, glucose-lowering, or lipid-modifying medications was not consistently available in the retrospective analytical dataset. Consequently, cardiometabolic criteria were operationalized using the available measured anthropometric and laboratory values rather than medication use. This may have resulted in some misclassification among treated participants whose measured values were below the corresponding thresholds and is acknowledged as a limitation.

Using this operational definition, 3,552 of the 7,011 participants (50.7%) were classified as having MASLD.

### 2.3 Assessment of cardiometabolic burden

A separate cardiometabolic burden score was constructed to characterize the cumulative clustering of metabolic abnormalities in the study population. This score was used as a secondary exposure and was conceptually distinct from the cardiometabolic criteria used to define MASLD.

Five cardiometabolic abnormalities were included:

1. Adiposity: BMI ≥24 kg/m2;
2. Elevated blood pressure: systolic blood pressure ≥140 mmHg or diastolic blood pressure ≥90 mmHg;
3. Dysglycemia: fasting plasma glucose ≥6.1 mmol/L or HbA1c ≥ 5.7%;
4. Hypertriglyceridemia: triglycerides ≥1.70 mmol/L;
5. Low HDL-C: HDL-C < 1.0 mmol/L in men or <1.3 mmol/L in women.

One point was assigned for each abnormal component, yielding a cardiometabolic burden score ranging from 0 to 5, with higher scores indicating a greater cumulative burden of measured cardiometabolic abnormalities.

For descriptive analyses, participants were categorized as having:

- low cardiometabolic burden: 0–1 abnormality;
- moderate cardiometabolic burden: 2–3 abnormalities;
- high cardiometabolic burden: 4–5 abnormalities.

The score was analyzed primarily as a continuous variable in regression analyses to preserve information and evaluate the association per additional cardiometabolic abnormality.

Importantly, because the burden score was constructed from adiposity, blood pressure, glycemic status, triglycerides, and HDL-C, these constituent variables were not simultaneously included as covariates in regression models in which the cardiometabolic burden score was the exposure. This approach was adopted to avoid conditioning on the components used to define the exposure itself and to improve interpretability of the estimated association.

The cardiometabolic burden score should therefore be interpreted as a study-specific measure of cumulative metabolic abnormalities rather than as a diagnostic criterion for MASLD or a validated causal mediator.

### 2.4 Assessment of urinary tubular biomarkers

Urinary tubular biomarkers were measured as part of routine clinical laboratory testing during the same health-examination episode. Four biomarkers were evaluated: urinary N-acetyl-*β*-D-glucosaminidase (uNAG), urinary *α*1-microglobulin (uA1M), urinary *β*2-microglobulin (u*β*2M), and urinary retinol-binding protein (uRBP).

These biomarkers were interpreted according to their distinct physiological characteristics rather than as interchangeable measures of structural tubular injury. uNAG, a lysosomal enzyme enriched in proximal tubular epithelial cells, was considered primarily a marker of tubular epithelial stress or cellular injury. In contrast, uA1M, u*β*2M, and uRBP are low-molecular-weight proteins that are normally filtered at the glomerulus and subsequently reabsorbed by proximal tubular epithelial cells; increased urinary concentrations therefore predominantly reflect impaired proximal tubular reabsorptive function.

The biomarkers were analyzed in their routinely reported units: U/L for uNAG and mg/L for uA1M, u*β*2M, and uRBP. For the primary categorical analyses, biomarker abnormalities were operationally defined as:

- uNAG >12 U/L;
- uA1M > 12 mg/L;
- u*β*2M > 0.30 mg/L; and
- uRBP >0.50 mg/L.

Because urinary reference intervals may vary according to assay platform, population characteristics, and urine concentration, these thresholds were treated as operational study cutoffs rather than universal diagnostic reference limits.

The original laboratory records included some results reported below the quantitative reporting range rather than as exact numerical concentrations. Specifically, 132 uNAG measurements were reported as <1.0 U/L, 2,188 uA1M measurements as <2.0 mg/L, 2,187 u*β*2M measurements as below the reported lower range (predominantly <0.1 mg/L), and 3,858 uRBP measurements as below the reported lower range (predominantly <0.1 mg/L). These observations were distinguished from conventional missing values. Because all such values were below the prespecified abnormality thresholds, they were classified as non-abnormal for categorical analyses. The handling of these left-censored observations in analyses of continuous biomarker concentrations is described in the Statistical Analysis section.

Urinary creatinine was not available for the vast majority of participants in the analytical cohort; therefore, urinary biomarker concentrations could not be normalized to urinary creatinine. The analyses consequently used the laboratory-reported urinary concentrations. This limitation was considered when interpreting between-participant differences in biomarker levels.

The urinary measurements were obtained from the hospital clinical laboratory under routine clinical testing procedures. Because the study was retrospective, complete specimen-level metadata regarding collection timing, transport interval, centrifugation, storage conditions, and assay-specific analyzer and reagent information were not consistently retained in the research dataset and were therefore not retrospectively reconstructed.

### 2.5 Definition of the primary and secondary urinary biomarker outcomes

The primary outcome was a composite urinary tubular biomarker abnormality, defined as the presence of at least one abnormal result among uNAG, uA1M, u*β*2M, and uRBP according to the prespecified operational cutoffs described above. This composite was intended to provide a sensitive summary phenotype of urinary tubular biomarker abnormalities and was not interpreted as histologically confirmed tubular injury.

Secondary outcomes included abnormalities in each individual urinary biomarker and the continuous concentrations of uNAG, uA1M, u*β*2M, and uRBP. A more stringent composite outcome, defined as abnormalities in two or more of the four urinary biomarkers, was evaluated in sensitivity analyses to reduce the influence of isolated abnormal measurements.

In the study population, 1,851 of 7,011 participants (26.4%) had at least one abnormal urinary biomarker, and 681 (9.7%) had abnormalities in at least two biomarkers. Individual abnormalities were observed in 1,080 participants (15.4%) for uNAG, 659 (9.4%) for uA1M, 932 (13.3%) for u*β*2M, and 191 (2.7%) for uRBP.

### 2.6 Statistical analysis

Continuous variables were examined for distributional characteristics before analysis. Approximately normally distributed variables were summarized as mean ± standard deviation, whereas skewed variables were presented as median and interquartile range. Categorical variables were summarized as numbers and percentages. Between-group comparisons were performed using Student's *t* test or one-way analysis of variance for approximately normally distributed continuous variables, the Mann–Whitney *U* test or Kruskal–Wallis test for skewed continuous variables, and the chi-square test for categorical variables, as appropriate. All tests were two-sided.

#### 2.6.1 Association between MASLD and the composite urinary biomarker outcome

Multivariable logistic regression was used to examine the association between MASLD and the primary composite urinary tubular biomarker abnormality. Odds ratios (ORs) and 95% confidence intervals (CIs) were reported. Because the study was cross-sectional, ORs were interpreted as measures of association with the prevalence of urinary biomarker abnormalities rather than as risk ratios or prospective risk estimates.

To characterize how the estimated MASLD association changed after conditioning on additional measured characteristics, sequential models were specified as follows:

- Model 1: adjusted for age and sex;
- Model 2: additionally adjusted for BMI, systolic blood pressure, and diastolic blood pressure;
- Model 3: additionally adjusted for fasting plasma glucose, HbA1c, triglycerides, HDL-C, LDL-C, and uric acid;
- Model 4: additionally adjusted for alanine aminotransferase, aspartate aminotransferase, gamma-glutamyl transferase, and eGFR.

Because several cardiometabolic variables included in Models 2 and 3 overlap with the operational definition of MASLD, these sequential models were interpreted as conditional association models rather than as estimates of a causal direct effect. Attenuation of the MASLD coefficient after covariate adjustment was not interpreted as evidence of mediation.

#### 2.6.2 Association between cardiometabolic burden and urinary biomarker abnormalities

Cardiometabolic burden was analyzed separately from MASLD. The burden score was modeled primarily as a continuous variable, with the OR interpreted per additional cardiometabolic abnormality.

The prespecified models were:

- Crude model: cardiometabolic burden score only;
- Model 1: adjusted for age and sex;
- Sensitivity model: additionally adjusted for eGFR.

Variables used to construct the cardiometabolic burden score—including BMI, blood pressure, glycemic measures, triglycerides, and HDL-C—were not included as covariates in models in which the burden score was the exposure, because such adjustment would condition on the constituent information used to define the exposure itself.

For descriptive purposes, cardiometabolic burden was also examined across low (0–1), moderate (2–3), and high (4–5) burden categories, with tests for trend performed using the continuous score where appropriate.

#### 2.6.3 Continuous urinary biomarker analyses

The distributions of urinary biomarker concentrations were right-skewed. In addition, a substantial proportion of uA1M, u*β*2M, and uRBP measurements, and a smaller proportion of uNAG measurements, were reported as being below the laboratory quantitative reporting limit rather than as exact concentrations.

Accordingly, values reported below the quantitative reporting limit were treated as left-censored observations rather than conventional missing values. For the primary analyses of continuous biomarker concentrations, censored regression models assuming a log-normal distribution were used, with observations censored at their corresponding reported laboratory limit. This approach allowed participants with below-reporting-limit results to contribute information without assigning arbitrary numerical concentrations.

As a sensitivity analysis, analyses were repeated among participants with exact quantitative biomarker measurements. Quantifiable biomarker concentrations were natural-log transformed, and multivariable linear regression was performed. Heteroskedasticity-consistent HC3 robust standard errors were used for statistical inference. For log-transformed outcomes, regression coefficients were additionally expressed as approximate percentage differences in geometric mean concentrations using: 100 × [exp(*β*)−1]% to facilitate clinical interpretation.

#### 2.6.4 Model diagnostics

Multicollinearity was evaluated using variance inflation factors (VIFs). A VIF <5 was considered to indicate the absence of substantial multicollinearity. For linear regression models, residual-versus-fitted plots and quantile–quantile plots were examined to assess functional form, heteroskedasticity, and residual distribution. Influential observations were evaluated using standard regression influence diagnostics.

For logistic regression models, the functional form of continuous covariates was examined graphically and by comparing linear specifications with flexible nonlinear specifications where appropriate. Model fit and the stability of effect estimates were evaluated across sequential adjustment sets.

#### 2.6.5 Subgroup and interaction analyses

Exploratory subgroup analyses of the association between MASLD and the primary composite outcome were conducted according to sex, age (<60 versus ≥60 years), BMI category, dysglycemia status, hypertension status, and eGFR category (<90 versus ≥90 mL/min/1.73 m^2^).

Effect modification was assessed using formal interaction terms between MASLD and the subgroup variable. Interpretation of heterogeneity was based on the *P*-value for interaction, rather than on whether the association reached statistical significance within one stratum but not another.

In stratified analyses, the variable defining the subgroup was not simultaneously included as an adjustment covariate. Where a subgroup was defined by multiple closely related measurements, the corresponding defining variables were omitted from the stratum-specific adjustment set to avoid inappropriate conditioning.

Because multiple interaction tests were performed, the Benjamini–Hochberg false-discovery-rate (FDR) procedure was applied across the prespecified interaction tests. Subgroup analyses were considered exploratory, and effect estimates with 95% CIs were emphasized alongside nominal and multiplicity-adjusted *P*-values.

#### 2.6.6 Sensitivity analyses

Several sensitivity analyses were performed to evaluate the robustness of the findings.

First, analyses were repeated after excluding participants with eGFR <60 mL/min/1.73 m^2^ to reduce the influence of established kidney dysfunction.

Second, analyses were repeated after excluding participants meeting diabetes-range glycemic criteria to assess whether the results were driven predominantly by more severe glucose dysregulation.

Third, a stricter composite urinary biomarker outcome requiring abnormalities in at least two of the four urinary biomarkers was evaluated.

Fourth, urinary biomarker abnormalities were alternatively defined using study-specific 90th-percentile thresholds.

Finally, results from the censored continuous-biomarker models were compared with complete-quantification analyses using log-transformed biomarker concentrations and HC3 robust standard errors.

A two-sided *P*-value <0.05 was considered statistically significant for the primary analysis. Secondary and exploratory analyses were interpreted with consideration of effect size, 95% CI, and multiplicity-adjusted results rather than nominal *P*-values alone. Statistical analyses were conducted using R version 4.3.0.

## 3 Results

### 3.1 Participant characteristics

Flow diagram illustrating the inclusion and exclusion criteria used to derive the final analytical cohort. After exclusion of participants with duplicate records or incomplete key measurements, 7,011 participants were included in the final analyses. No missing values were present for covariates included in the final regression models.

The participant selection process is shown in Figure 1. Among the 7,011 participants included in the final analysis, the mean age was 55.6 ± 11.2 years, and 2,872 (41.0%) were male. No missing values were present for the covariates included in the final analytical models. Participants were categorized according to cardiometabolic burden score, with 3,027 (43.2%) classified as low burden, 3,165 (45.1%) as moderate burden, and 819 (11.7%) as high burden.

**Figure 1:**
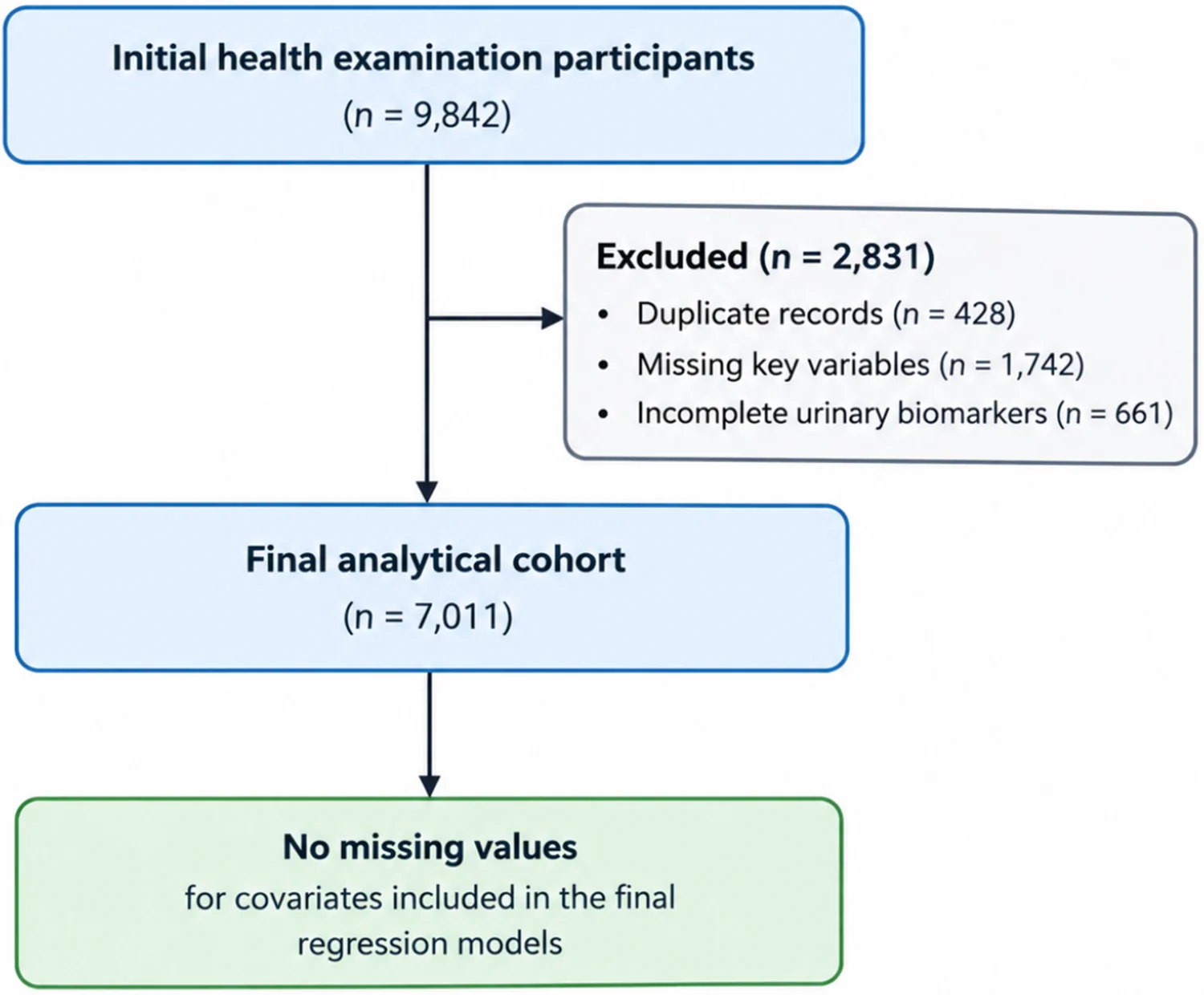
Flow diagram of participant selection.

Participants with greater cardiometabolic burden were generally older and had less favorable metabolic profiles. Across increasing burden categories, BMI, systolic and diastolic blood pressure, fasting plasma glucose, HbA1c, triglycerides, uric acid, and liver enzyme concentrations increased, whereas HDL-C concentrations decreased (Table 1). Although eGFR also differed statistically across burden categories, the absolute between-group differences were modest and should not be interpreted as evidence of clinically meaningful kidney dysfunction solely on the basis of statistical significance.

**Table 1:** Characteristics of the study population according to cardiometabolic burden.

Characteristic	Overall (*n* = 7,011)	Low burden (0–1) (*n* = 3,027)	Moderate burden (2–3) (*n* = 3,165)	High burden (4–5) (*n* = 819)	*P*-value
Age, years	55.59 ± 11.20	52.57 ± 11.90	57.83 ± 10.10	58.05 ± 9.83	<0.001
Male sex, *n* (%)	2,872 (41.0)	885 (29.2)	1,555 (49.1)	432 (52.7)	<0.001
BMI, kg/m^2^	24.37 ± 3.23	22.38 ± 2.48	25.53 ± 2.86	27.20 ± 2.70	<0.001
SBP, mmHg	124.98 ± 17.63	116.54 ± 13.92	129.39 ± 17.00	139.15 ± 16.95	<0.001
DBP, mmHg	73.41 ± 11.28	68.81 ± 9.64	75.75 ± 10.91	81.34 ± 11.16	<0.001
Fasting glucose, mmol/L	5.14 (4.81–5.62)	4.92 (4.66–5.22)	5.31 (4.97–5.84)	5.69 (5.16–6.62)	<0.001
HbA1c, %	5.70 (5.50–6.00)	5.50 (5.30–5.70)	5.80 (5.60–6.20)	6.00 (5.80–6.50)	<0.001
Total cholesterol, mmol/L	5.01 (4.38–5.67)	5.00 (4.43–5.64)	5.03 (4.36–5.71)	4.95 (4.29–5.63)	0.075
Triglycerides, mmol/L	1.28 (0.90–1.90)	0.97 (0.73–1.28)	1.50 (1.09–2.10)	2.49 (1.95–3.58)	<0.001
HDL-C, mmol/L	1.40 (1.17–1.65)	1.57 (1.38–1.81)	1.31 (1.14–1.52)	1.06 (0.91–1.23)	<0.001
LDL-C, mmol/L	3.14 (2.56–3.74)	3.08 (2.54–3.66)	3.21 (2.63–3.83)	3.06 (2.51–3.69)	<0.001
Uric acid, μmol/L	328.68 ± 81.15	299.63 ± 72.32	344.78 ± 79.33	373.81 ± 81.09	<0.001
ALT, U/L	20 (15–28)	17 (13–23)	21 (16–29)	26 (19–37)	<0.001
AST, U/L	21 (18–25)	21 (18–24)	21 (18–25)	22 (19–28)	<0.001
GGT, U/L	22 (15–34)	17 (13–25)	25 (17–39)	31 (22–48)	<0.001
Creatinine, μmol/L	62.15 ± 15.38	59.94 ± 13.78	63.59 ± 15.87	64.76 ± 17.80	<0.001
eGFR, mL/min/1.73 m^2^	109.80 ± 22.33	111.30 ± 21.59	108.68 ± 22.38	108.61 ± 24.49	<0.001
Hepatic steatosis, *n* (%)	3,684 (52.5)	913 (30.2)	2,074 (65.5)	697 (85.1)	<0.001
MASLD, *n* (%)	3,552 (50.7)	-	-	-	-
uA1M, mg/L	3.67 (2.00–7.21)	2.81 (2.00–5.74)	4.18 (2.00–7.89)	5.14 (2.30–9.85)	<0.001
uNAG, U/L	6.23 (3.92–9.66)	5.42 (3.38–8.41)	6.75 (4.32–10.29)	7.88 (4.98–12.34)	<0.001
u*β*2M, mg/L	0.15 (0.10–0.23)	0.15 (0.10–0.22)	0.15 (0.10–0.23)	0.14 (0.10–0.26)	0.424
uRBP, mg/L	0.10 (0.10–0.16)	0.10 (0.10–0.14)	0.10 (0.10–0.17)	0.11 (0.10–0.21)	<0.001

Values are presented as mean ± standard deviation, median (interquartile range), or number (%), as appropriate. Cardiometabolic burden was calculated from five abnormalities: BMI ≥24 kg/m^2^, elevated blood pressure (SBP ≥140 mmHg or DBP ≥90 mmHg), dysglycemia (fasting plasma glucose ≥6.1 mmol/L or HbA1c ≥ 5.7%), triglycerides ≥1.70 mmol/L, and low HDL-C (<1.0 mmol/L in men or <1.3 mmol/L in women). *P*-values were calculated using one-way analysis of variance, Kruskal–Wallis tests, or chi-square tests, as appropriate. Statistical significance does not necessarily imply clinical importance.

The prevalence of ultrasonographically detected hepatic steatosis increased markedly with cardiometabolic burden, from 30.2% in the low-burden group to 65.5% in the moderate-burden group and 85.1% in the high-burden group. Overall, 3,552 of 7,011 participants (50.7%) were classified as having MASLD after verification of the classification algorithm.

Urinary biomarker concentrations also showed heterogeneous patterns across cardiometabolic burden categories. Median uNAG increased from 5.42 in the low-burden group to 6.75 in the moderate-burden group and 7.88 U/L in the high-burden group. Similar increases were observed for uA1M and uRBP, whereas median u*β*2M concentrations were similar across the three groups. The exact Kruskal–Wallis *P*-value for u*β*2M was 0.424, replacing the previously reported “NS.”

### 3.2 Prevalence of urinary tubular biomarker abnormalities

Overall, 1,851 of 7,011 participants (26.4%) had at least one abnormal urinary tubular biomarker according to the prespecified operational cutoffs. A more stringent phenotype, defined as abnormalities in at least two urinary biomarkers, was present in 681 participants (9.7%).

Among the individual biomarkers, abnormal uNAG was the most frequent finding, observed in 1,080 participants (15.4%), followed by abnormal u*β*2M in 932 (13.3%), abnormal uA1M in 659 (9.4%), and abnormal uRBP in 191 (2.7%).

The prevalence of urinary biomarker abnormalities was generally higher among participants with MASLD than among those without MASLD. The primary composite abnormality was present in 1,090 of 3,552 participants with MASLD (30.7%), compared with 761 of 3,459 participants without MASLD (22.0%). Similarly, abnormalities in at least two urinary biomarkers were observed in 427 participants with MASLD (12.0%) and 254 participants without MASLD (7.3%).

For the individual biomarkers, abnormal uNAG was observed in 19.5% of participants with MASLD and 11.2% of those without MASLD; abnormal uA1M in 11.8% versus 7.0%; abnormal u*β*2M in 14.0% versus 12.6%; and abnormal uRBP in 3.5% versus 2.0%, respectively (Table 2).

**Table 2:** Prevalence of urinary tubular biomarker abnormalities according to MASLD status.

Urinary biomarker outcome	Overall (*n* = 7,011)	Non-MASLD (*n* = 3,459)	MASLD (*n* = 3,552)
≥1 abnormal biomarker	1,851 (26.4%)	761 (22.0%)	1,090 (30.7%)
Abnormal uNAG	1,080 (15.4%)	388 (11.2%)	692 (19.5%)
Abnormal uA1M	659 (9.4%)	241 (7.0%)	418 (11.8%)
Abnormal uβ2M	932 (13.3%)	435 (12.6%)	497 (14.0%)
Abnormal uRBP	191 (2.7%)	68 (2.0%)	123 (3.5%)
≥2 abnormal biomarkers	681 (9.7%)	254 (7.3%)	427 (12.0%)

Values are presented as number (%). Urinary biomarker abnormalities were defined using the prespecified operational cutoffs: uNAG >12 U/L, uA1M > 12 mg/L, uβ2M > 0.30 mg/L, and uRBP >0.50 mg/L. The primary composite outcome was defined as at least one abnormal urinary biomarker. These thresholds were used as operational study definitions rather than universal diagnostic reference limits.

These descriptive differences were subsequently evaluated using multivariable regression models to determine how the MASLD association changed after sequential adjustment for measured demographic, cardiometabolic, hepatic, and renal characteristics.

### 3.3 Association between MASLD and the composite urinary tubular biomarker abnormality

In age- and sex-adjusted analyses, MASLD was associated with higher odds of the composite urinary tubular biomarker abnormality (OR, **1.24**; 95% CI, **1.11–1.39**; *P* < 0.001) (Table 3). After additional adjustment for BMI and blood pressure, the association was attenuated but remained statistically significant (OR, **1.15**; 95% CI, **1.01–1.30**; *P* = 0.031).

**Table 3:** Association between MASLD and the composite urinary tubular biomarker abnormality.

Model	Adjustment	OR (95% CI)	*P*-value
Model 1	Age, sex	1.24 (1.11–1.39)	<0.001
Model 2	+ BMI, SBP, DBP	1.15 (1.01–1.30)	0.031
Model 3	+ FPG, HbA1c, TG, HDL-C, LDL-C, uric acid	1.02 (0.90–1.17)	0.717
Model 4	+ ALT, AST, GGT, eGFR	1.02 (0.89–1.17)	0.785

Odds ratios were estimated using multivariable logistic regression. The outcome was the composite urinary tubular biomarker abnormality, defined as at least one abnormal result among uNAG, uA1M, uβ2M, and uRBP. Because the study was cross-sectional, ORs represent associations with the prevalence of the outcome rather than prospective risk. Several cardiometabolic covariates overlap with the operational definition of MASLD; therefore, attenuation across sequential models should not be interpreted as evidence of mediation.

With further adjustment for fasting plasma glucose, HbA1c, triglycerides, HDL-C, LDL-C, and uric acid, the estimated association was substantially reduced and was no longer statistically significant (OR, **1.02**; 95% CI, **0.90–1.17**; *P* = 0.717). Additional adjustment for liver enzymes and eGFR produced little further change in the estimate (OR, **1.02**; 95% CI, **0.89–1.17**; *P* = 0.785).

These sequential models indicate that the observed association between MASLD and the composite urinary biomarker phenotype was markedly attenuated after conditioning on measured cardiometabolic characteristics. Because several of these covariates overlap with the operational definition of MASLD, the attenuation should not be interpreted as evidence of formal mediation or as proof that hepatic steatosis has no independent biological relevance.

### 3.4 Association between cardiometabolic burden and the composite urinary tubular biomarker abnormality

Greater cardiometabolic burden was consistently associated with higher odds of the composite urinary tubular biomarker abnormality (Table 4). In the crude model, each one-point increase in the cardiometabolic burden score was associated with a **33% higher odds** of having at least one abnormal urinary biomarker (OR, **1.33**; 95% CI, **1.28–1.39**; *P* < 0.001).

**Table 4:** Association between cardiometabolic burden and the composite urinary tubular biomarker abnormality.

Model	Adjustment	OR per 1-point increase (95% CI)	*P*-value
Crude model	None	1.33 (1.28–1.39)	<0.001
Model 1	Age, sex	1.21 (1.16–1.26)	<0.001
Sensitivity model	Age, sex, eGFR	1.22 (1.16–1.27)	<0.001

Odds ratios were estimated using logistic regression and represent the association per one-point increase in the cardiometabolic burden score. The primary outcome was the presence of at least one abnormal urinary biomarker. Variables used to construct the burden score—including BMI, blood pressure, glycemic measures, triglycerides, and HDL-C—were not included as covariates in these models. Because the study was cross-sectional, ORs represent associations with prevalent urinary biomarker abnormalities rather than prospective risk.

After adjustment for age and sex, the association was attenuated but remained statistically significant (OR, **1.21**; 95% CI, **1.16–1.26**; *P* < 0.001). Additional adjustment for eGFR produced little change in the estimate (OR, **1.22**; 95% CI, **1.16–1.27**; *P* < 0.001).

Unlike the original analysis, BMI, blood pressure, glycemic measures, triglycerides, and HDL-C were not included as covariates in these models because these variables were used to construct the cardiometabolic burden score itself. The revised analysis therefore estimates the association between cumulative cardiometabolic abnormalities and the urinary biomarker phenotype without conditioning on the constituent components of the exposure.

### 3.5 Association between cardiometabolic burden and individual urinary tubular biomarkers

Greater cardiometabolic burden was also associated with higher concentrations of individual urinary tubular biomarkers (Table 5). In age- and sex-adjusted analyses restricted to participants with quantitatively reported biomarker concentrations, each one-point increase in the cardiometabolic burden score was associated with higher concentrations of all four urinary biomarkers.

**Table 5:** Association between cardiometabolic burden and individual urinary tubular biomarker concentrations.

Outcome	N	β per 1-point increase	95% CI	Approximate difference in geometric mean	*P*-value
log(uNAG)	6,879	0.0679	0.0552 to 0.0806	+7.0%	<0.001
log(uA1M)	4,823	0.0540	0.0395 to 0.0685	+5.5%	<0.001
log(uβ2M)	4,824	0.0205	0.0067 to 0.0344	+2.1%	0.004
log(uRBP)	3,153	0.0429	0.0249 to 0.0609	+4.4%	<0.001

β coefficients and 95% confidence intervals were estimated using multivariable linear regression with HC3 heteroskedasticity-consistent standard errors. Models were adjusted for age and sex. Biomarker concentrations were natural-log transformed. Approximate percentage differences in geometric mean concentrations were calculated as 100 × [exp(β)−1]%. Variables used to construct the cardiometabolic burden score were not included as covariates. Analyses were restricted to observations with quantitatively reported biomarker concentrations; measurements reported below the laboratory quantitative reporting limit were evaluated separately in censored-data analyses.

For uNAG, each additional cardiometabolic abnormality was associated with a **0.0679-unit increase in log-transformed concentration** (95% CI, 0.0552–0.0806; *P* < 0.001), corresponding to an approximately **7.0% higher geometric mean uNAG concentration**. The corresponding estimate for uA1M was *β*=**0.0540** (95% CI, 0.0395–0.0685; *P* < 0.001), equivalent to an approximately **5.5% higher geometric mean concentration** per one-point increase in cardiometabolic burden.

Associations were smaller but remained statistically significant for u*β*2M (*β*=**0.0205**; 95% CI, 0.0067–0.0344; *P* = 0.004), corresponding to an approximately **2.1% higher geometric mean concentration**, and for uRBP (*β*=**0.0429**; 95% CI, 0.0249–0.0609; *P* < 0.001), corresponding to an approximately **4.4% higher geometric mean concentration**.

These associations were estimated without adjustment for BMI, blood pressure, glycemic measures, triglycerides, or HDL-C because these variables constitute the cardiometabolic burden score itself. HC3 heteroskedasticity-consistent standard errors were used to reduce sensitivity of statistical inference to heteroskedasticity. The direction of the associations was consistent across all four biomarkers, although the magnitude differed according to the biomarker examined.

### 3.6 Subgroup and interaction analyses

Exploratory subgroup analyses were performed to assess whether the association between MASLD and the composite urinary tubular biomarker abnormality differed according to sex, age, BMI category, dysglycemia status, hypertension status, and eGFR category (Table 6). Interpretation of heterogeneity was based on formal interaction tests rather than on the statistical significance of individual stratum-specific estimates.

**Table 6:** Subgroup analyses of the association between MASLD and the composite urinary tubular biomarker abnormality.

Subgroup	Category	OR (95% CI)	*P*-value	P for interaction	FDR-adjusted *q*-value
Sex	Male	1.04 (0.86–1.25)	0.695	0.374	0.374
	Female	0.99 (0.81–1.20)	0.906		
Age	<60 years	1.20 (1.00–1.44)	0.054	0.017	**0**.**034**
	≥60 years	0.87 (0.71–1.07)	0.187		
BMI	<24 kg/m^2^	0.85 (0.70–1.05)	0.135	0.017	**0**.**034**
	≥24 kg/m^2^	1.15 (0.96–1.37)	0.140		
Dysglycemia	No	0.84 (0.66–1.07)	0.150	<0.001	**<0**.**006**
	Yes	1.23 (1.05–1.46)	0.013		
Hypertension	No	1.09 (0.91–1.32)	0.346	0.188	0.226
	Yes	0.96 (0.78–1.16)	0.648		
eGFR	<90 mL/min/1.73 m^2^	0.72 (0.54–0.96)	0.025	0.043	0.065
	≥90 mL/min/1.73 m^2^	1.13 (0.97–1.32)	0.121		

Odds ratios and 95% confidence intervals were estimated using multivariable logistic regression. The variable defining each subgroup was omitted from the corresponding stratum-specific adjustment model. Heterogeneity was assessed using formal interaction tests. Benjamini–Hochberg false-discovery-rate correction was applied across the six prespecified interaction tests. Subgroup analyses were exploratory.

Bold values indicate statistically significant interactions after Benjamini-Hochberg false-discovery-rate correction (FDR-adjusted q-value < 0.05).

No evidence of interaction was observed according to sex (P for interaction = 0.374) or hypertension status (P for interaction = 0.188). In contrast, interaction tests suggested heterogeneity according to age (P for interaction = 0.017), BMI category (P for interaction = 0.017), dysglycemia status (P for interaction <0.001), and eGFR category (P for interaction = 0.043).

The most pronounced heterogeneity was observed according to dysglycemia status. Among participants with dysglycemia, MASLD was associated with higher odds of the composite urinary biomarker abnormality (OR, **1.23**; 95% CI, **1.05–1.46**; *P* = 0.013), whereas the corresponding estimate among participants without dysglycemia was OR **0.84** (95% CI, **0.66–1.07**; *P* = 0.150). Importantly, this contrast was interpreted on the basis of the significant interaction test rather than the difference in within-stratum statistical significance.

Interaction tests also suggested heterogeneity by age and BMI category. Among participants aged <60 years, the estimated association was OR **1.20** (95% CI, **1.00–1.44**), compared with OR **0.87** (95% CI, **0.71–1.07**) among those aged ≥60 years. For BMI, the corresponding estimates were OR **0.85** (95% CI, **0.70–1.05**) among participants with BMI <24 kg/m^2^ and OR **1.15** (95% CI, **0.96–1.37**) among those with BMI ≥24 kg/m^2^. Neither pair of stratum-specific estimates should be interpreted solely on the basis of whether an individual *P*-value crossed the conventional 0.05 threshold.

For eGFR, the interaction test was nominally significant (P for interaction = 0.043). Unexpectedly, among participants with eGFR <90 mL/min/1.73 m^2^, MASLD was inversely associated with the composite urinary biomarker abnormality (OR, **0.72**; 95% CI, **0.54–0.96**; *P* = 0.025), whereas the corresponding estimate among those with eGFR ≥90 mL/min/1.73 m^2^ was OR **1.13** (95% CI, **0.97–1.32**; *P* = 0.121). Given the unexpected direction and exploratory nature of this finding, it should be interpreted cautiously.

After application of the Benjamini–Hochberg false-discovery-rate procedure across the six prespecified interaction tests, the interactions for **dysglycemia, age, and BMI category remained statistically significant**, whereas the interaction for eGFR no longer met the FDR-adjusted significance threshold. The sex and hypertension interactions remained nonsignificant. These subgroup findings should therefore be regarded as exploratory and hypothesis-generating.

### 3.7 Sensitivity analyses

The association between MASLD and the composite urinary tubular biomarker abnormality was further examined in several sensitivity analyses (Table 7).

**Table 7:** Sensitivity analyses of the association between MASLD and urinary tubular biomarker abnormalities.

Sensitivity analysis	Participants, n	Fully adjusted OR (95% CI)	*P*-value
Main analysis	7,011	1.02 (0.89–1.17)	0.785
Excluding participants with eGFR <60 mL/min/1.73 m^2^	6,958	1.04 (0.91–1.20)	0.543
Excluding participants with diabetes-like glycemic status	6,138	1.02 (0.88–1.18)	0.820
Stricter composite outcome: ≥2 abnormal biomarkers	7,011	0.97 (0.79–1.19)	0.755
90th-percentile biomarker thresholds	7,011	1.07 (0.93–1.22)	0.329

Odds ratios and 95% confidence intervals were estimated using multivariable logistic regression. The fully adjusted models included age, sex, BMI, systolic and diastolic blood pressure, fasting plasma glucose, HbA1c, triglycerides, HDL-C, LDL-C, uric acid, ALT, AST, GGT, and eGFR, except where the sensitivity-analysis design required modification of the adjustment set. The stricter composite outcome was defined as abnormalities in at least two urinary biomarkers. Because the study was cross-sectional, ORs are interpreted as associations with prevalent urinary biomarker abnormalities rather than prospective risk.

First, excluding participants with eGFR <60 mL/min/1.73 m^2^ yielded an effect estimate similar to that observed in the primary fully adjusted analysis (OR, **1.04**; 95% CI, **0.91–1.20**; *P* = 0.543). Similarly, after excluding participants meeting the prespecified diabetes-like glycemic criterion, the association remained close to the null (OR, **1.02**; 95% CI, **0.88–1.18**; *P* = 0.820).

Second, when the urinary biomarker phenotype was defined more stringently as abnormalities in at least **two of the four urinary biomarkers**, MASLD was not significantly associated with the outcome after multivariable adjustment (OR, **0.97**; 95% CI, **0.79–1.19**; *P* = 0.755).

Third, when abnormal urinary biomarker concentrations were alternatively defined using study-specific **90th-percentile thresholds**, the estimated association remained nonsignificant (OR, **1.07**; 95% CI, **0.93–1.22**; *P* = 0.329).

Overall, the point estimates across these alternative sample restrictions and outcome definitions remained close to 1.0, indicating that the null association observed in the most extensively adjusted primary model was not materially altered by these alternative analytical specifications.

## 4 Discussion

In this cross-sectional health-examination study of 7,011 adults, we examined the associations of MASLD and cumulative cardiometabolic burden with a composite urinary tubular biomarker abnormality. Three findings merit emphasis. First, MASLD was associated with a higher prevalence of the composite urinary biomarker phenotype after adjustment for age and sex, but this association was markedly attenuated after further conditioning on measured cardiometabolic characteristics and remained close to the null after additional adjustment for liver enzymes and eGFR. Second, when cardiometabolic burden was analyzed as a separate exposure without adjustment for its constituent components, greater burden was consistently associated with higher odds of the composite urinary biomarker abnormality and with higher concentrations of uNAG, uA1M, u*β*2M, and uRBP. Third, exploratory interaction analyses suggested potential heterogeneity according to dysglycemia, age, and BMI, with the dysglycemia interaction showing the most pronounced difference. Taken together, these findings suggest substantial statistical overlap between MASLD and cardiometabolic abnormalities in relation to urinary tubular biomarker phenotypes, while avoiding the interpretation that cardiometabolic burden formally mediates the association.

Our findings should be interpreted in the context of a substantial literature linking steatotic liver disease with kidney disease. Previous cohort studies and meta-analyses have consistently reported associations of NAFLD with prevalent and incident CKD, albuminuria, and reduced eGFR (11–15). More recent studies using MAFLD or MASLD-related definitions have generally supported an association between metabolically defined steatotic liver disease and CKD (16, 17). These prior findings have contributed to the concept that metabolic liver disease and kidney dysfunction may share common systemic pathways, including insulin resistance, chronic low-grade inflammation, oxidative stress, endothelial dysfunction, and altered lipid metabolism (8–10, 18–21). However, the renal outcomes examined in most of these studies differ substantially from those evaluated here. Whereas previous studies have primarily focused on eGFR decline, albuminuria, or incident CKD, the present study examined a phenotype defined by urinary markers reflecting tubular epithelial stress and proximal tubular reabsorptive dysfunction. Therefore, the attenuation of the MASLD association in our fully adjusted model should not be viewed as contradicting the existing MASLD–CKD literature.

Several methodological differences may also explain why the fully adjusted MASLD estimate in our study was close to the null. First, our study was cross-sectional, whereas many prior studies reporting incident CKD used longitudinal designs (11–17). Second, exposure definitions have evolved from NAFLD to MAFLD and MASLD, with the current MASLD framework explicitly incorporating cardiometabolic criteria into the disease definition (1–3). Third, adjustment strategies differ considerably across studies. In the present analysis, several covariates introduced in the sequential models—such as adiposity, blood pressure, glucose regulation, triglycerides, and HDL-C—overlap directly with the operational definition of MASLD. Consequently, the attenuation from an age- and sex-adjusted OR of 1.24 to a fully adjusted OR of 1.02 indicates that the observed association was not independent of these measured cardiometabolic characteristics. It does not demonstrate that hepatic steatosis has no biological contribution to renal physiology, nor does it establish a causal mediating pathway.

This distinction is particularly important under the current MASLD nomenclature. Cardiometabolic dysfunction is not simply a potential confounder external to the disease definition; rather, it is an integral component of MASLD itself (1–3). Conditioning on these characteristics therefore estimates a different contrast from the minimally adjusted association. Sequential attenuation may arise from shared etiological factors, exposure-definition overlap, overadjustment, non-collapsibility of odds ratios, or other statistical mechanisms. For this reason, we interpret the sequential models descriptively and avoid referring to the attenuation as “mediation.” This interpretation is consistent with the reviewer's concern that MASLD and cumulative cardiometabolic burden are related but not statistically independent constructs.

The revised analysis of cardiometabolic burden provides complementary information. In contrast to the original analysis, the burden score was not adjusted for BMI, blood pressure, glycemic variables, triglycerides, or HDL-C because these variables were used to construct the score itself. Under this specification, each additional cardiometabolic abnormality was associated with approximately 21% higher odds of the composite urinary biomarker abnormality after adjustment for age and sex, and the estimate was essentially unchanged after additional adjustment for eGFR. This finding is consistent with previous studies showing that metabolic syndrome and increasing numbers of metabolic abnormalities are associated with greater CKD prevalence and incidence (31–33). Importantly, our results extend this literature from conventional kidney outcomes to urinary markers reflecting tubular physiology.

The continuous biomarker analyses further supported this pattern. Each additional cardiometabolic abnormality was associated with approximately 7.0% higher geometric mean uNAG, 5.5% higher uA1M, 2.1% higher u*β*2M, and 4.4% higher uRBP concentrations in age- and sex-adjusted analyses. Although these effect sizes were modest, their consistent direction across biologically distinct markers suggests that cumulative metabolic burden may be associated with multiple aspects of tubular physiology rather than with a single isolated laboratory abnormality. These findings should nevertheless be interpreted as associations rather than evidence of direct metabolic injury.

The biological interpretation of the individual biomarkers requires particular caution. uNAG is a lysosomal enzyme enriched in proximal tubular epithelial cells and has been used as a marker of tubular epithelial stress or injury in diabetic, toxic, and metabolic kidney disorders (22, 26, 27). By contrast, uA1M, u*β*2M, and uRBP are low-molecular-weight proteins whose urinary excretion is more directly related to impaired proximal tubular reabsorption (23, 24, 28–30). Thus, the composite outcome used in this study combines partially distinct physiological processes. It should therefore be viewed as a pragmatic urinary tubular biomarker phenotype rather than as a validated pathological entity. None of these biomarkers was validated against renal histology in the present study, and their concentrations may also be influenced by urine dilution, urinary pH, protein stability, assay characteristics, and specimen handling (23, 24, 28–30).

The observed associations between cardiometabolic burden and urinary biomarkers are biologically plausible in light of the high metabolic demands of the proximal tubule. Proximal tubular epithelial cells depend heavily on mitochondrial oxidative metabolism to support extensive solute reabsorption (20, 21, 25). Hyperglycemia may increase tubular glucose transport and metabolic workload, while insulin resistance, lipid overload, mitochondrial dysfunction, and oxidative stress can alter tubular cellular homeostasis (18–21, 25). These pathways provide a plausible biological framework for the positive associations observed in our study, but they were not directly measured and should therefore be regarded as mechanistic hypotheses rather than demonstrated pathways.

Among the exploratory subgroup analyses, the interaction with dysglycemia was the most notable. MASLD was associated with higher odds of the composite urinary biomarker abnormality among participants with dysglycemia, whereas the corresponding association was weaker among those without dysglycemia. This finding is biologically plausible because abnormal glucose metabolism is strongly implicated in proximal tubular dysfunction and diabetic kidney disease (19–23). Chronic hyperglycemia can increase proximal tubular glucose reabsorption, alter mitochondrial metabolism, promote oxidative stress, and activate inflammatory and fibrotic signaling pathways (19–23). Nevertheless, the present data do not establish that dysglycemia causally increases susceptibility to MASLD-associated tubular abnormalities. The analysis was cross-sectional and exploratory, multiple interactions were tested, and residual confounding remains possible. Therefore, this finding should be considered hypothesis-generating and requires confirmation in prospective studies.

Interaction tests also suggested heterogeneity according to age and BMI category, although the stratum-specific estimates were imprecise and neither pattern should be interpreted solely on the basis of whether one subgroup reached conventional statistical significance. The nominal interaction by eGFR was more difficult to interpret because the association among participants with eGFR <90 mL/min/1.73 m^2^ was unexpectedly inverse. After false-discovery-rate adjustment, this interaction no longer met the multiplicity-adjusted significance threshold. We therefore regard this result as exploratory and potentially attributable to chance, residual confounding, selection effects, or differences in urinary biomarker handling across levels of kidney function rather than as evidence of a protective association.

The sensitivity analyses further inform interpretation of the MASLD findings. Excluding participants with eGFR <60 mL/min/1.73 m^2^, excluding participants with more marked glycemic abnormalities, using a stricter outcome requiring at least two abnormal urinary biomarkers, and applying alternative percentile-based biomarker thresholds all yielded fully adjusted effect estimates close to 1.0. These analyses do not prove the absence of an association, but they indicate that the null estimate in the most extensively adjusted model was not driven solely by a single outcome definition or by participants with substantially reduced kidney function.

From a clinical perspective, the present findings should not be interpreted as supporting routine measurement of uNAG, uA1M, u*β*2M, or uRBP in all individuals with MASLD. Current MASLD management recommendations emphasize comprehensive assessment and treatment of cardiometabolic risk, including adiposity, dysglycemia, hypertension, and dyslipidemia (34, 35), while kidney disease guidelines continue to rely principally on established measures such as eGFR and albuminuria for clinical risk assessment (36). Our results instead support further evaluation of tubular biomarkers in prospective studies to determine whether they add prognostic information beyond conventional renal measures, particularly in individuals with substantial cardiometabolic burden.

### 4.1 Strengths and limitations

This study has several strengths. First, the relatively large sample of 7,011 unique health-examination participants provided sufficient precision for multivariable and exploratory subgroup analyses. Second, the availability of four urinary biomarkers allowed assessment of different aspects of tubular physiology rather than reliance on a single marker. Third, the revised analytical strategy separated MASLD from cumulative cardiometabolic burden and avoided adjusting the burden score for the variables used to construct it. Fourth, the analysis explicitly distinguished laboratory values reported below quantitative reporting limits from conventional missing data and incorporated sensitivity analyses addressing model assumptions and alternative outcome definitions.

Several limitations should also be acknowledged. First, the cross-sectional design precludes determination of temporal ordering and causal relationships. We cannot establish whether MASLD or cardiometabolic abnormalities preceded the urinary biomarker abnormalities, nor whether these abnormalities predict subsequent CKD. Second, the study was based on health-examination attendees at a single hospital rather than a population-based probability sample, limiting generalizability. Third, hepatic steatosis was assessed by ultrasonography, which has limited sensitivity for mild steatosis and cannot precisely quantify hepatic fat or characterize fibrosis. Device- and operator-specific metadata were not consistently retained in the retrospective dataset. Fourth, medication history was not systematically available, which may have led to misclassification of cardiometabolic criteria among treated participants whose measured values were controlled.

Fifth, urinary creatinine was unavailable for the vast majority of participants; consequently, urinary biomarker concentrations could not be normalized for urine concentration. Between-person differences in hydration and urine dilution may therefore have contributed to variability in biomarker measurements. In addition, complete specimen-level information regarding urine collection timing, transport, storage, and processing was not retained in the retrospective analytical dataset. Sixth, the operational biomarker thresholds used in this study should not be interpreted as universal diagnostic reference limits because assay-specific reference intervals may vary across laboratories and populations. Seventh, the composite outcome combined biomarkers with different biological interpretations and was not validated against renal histology. Finally, residual confounding from unmeasured factors such as smoking, alcohol intake, physical activity, dietary patterns, medication use, and other comorbid conditions cannot be excluded.

Despite these limitations, the present findings refine the interpretation of renal biomarker abnormalities in MASLD. Rather than supporting a simple causal pathway from hepatic steatosis through cardiometabolic burden to tubular injury, the data indicate substantial overlap among MASLD, cumulative cardiometabolic abnormalities, and urinary tubular biomarker phenotypes. Prospective studies incorporating standardized urine collection, creatinine-normalized biomarker measurements, more precise hepatic phenotyping, and repeated assessment of renal outcomes will be required to determine the temporal and prognostic significance of these associations.

## 5 Conclusion

In this cross-sectional health-examination cohort, MASLD was associated with a higher prevalence of a composite urinary tubular biomarker abnormality after adjustment for age and sex, but this association was markedly attenuated after further conditioning on measured cardiometabolic and renal characteristics. This attenuation indicates substantial statistical overlap between MASLD and accompanying cardiometabolic abnormalities but does not establish mediation, causality, or the absence of an independent biological contribution of hepatic steatosis.

In separate analyses, greater cumulative cardiometabolic burden was consistently associated with higher odds of the composite urinary biomarker abnormality and with higher concentrations of uNAG, uA1M, u*β*2M, and uRBP. These findings suggest that clustering of cardiometabolic abnormalities is closely associated with urinary tubular biomarker phenotypes in individuals undergoing routine health examinations.

Exploratory subgroup analyses suggested possible heterogeneity according to dysglycemia status, although these findings require confirmation. Prospective studies incorporating standardized urine collection, creatinine-normalized biomarker measurements, more precise hepatic phenotyping, and repeated assessment of kidney outcomes are needed to determine the temporal and prognostic significance of these associations and whether urinary tubular biomarkers provide information beyond established renal measures.

## Data Availability

The raw data supporting the conclusions of this article will be made available by the authors, without undue reservation.
